# Efficacy of bandage contact lens for the management of dry eye disease after cataract surgery

**DOI:** 10.1007/s10792-021-01692-6

**Published:** 2021-01-28

**Authors:** Xingdi Wu, Yajuan Ma, Xiang Chen, Suhong He, Xueqi Lin, Xuewen Yu, Weijie Chen, Chenqi Luo, Wen Xu

**Affiliations:** 1 Eye Center, Affiliated Second Hospital, School of Medicine, Zhejiang University, 88 Jiefang Road, Hangzhou, 310009 China; 2 Zhejiang Rongjun Hospital, Jiaxing, China; 3 Suichang Hospital of Traditional Chinese Medicine, Suichang, China; 4 Linhai First People’s Hospital, Linhai, China

**Keywords:** Cataract, Surgery, Dry eye disease, Bandage contact lens

## Abstract

**Purpose:**

We aimed to evaluate the efficacy of bandage contact lens (BCL) for the management of dry eye disease (DED) after cataract surgery.

**Methods:**

A total of 120 patients (140 eyes) with age-related cataract and DED were enrolled in this study. Patients underwent standard micro-incision phacoemulsification surgeries and were divided into control or BCL groups. Slit-lamp biomicroscopic examination, Ocular Surface Disease Index, keratograph analysis and Schirmer I test were executed, and the levels of tear inflammatory molecules were detected.

**Results:**

In the control group, the NIAvg-BUT and Schirmer I test scores were significantly decreased at 1 week post-operation compared with baseline levels (*P* = 0.035 and *P* = 0.009, respectively). In the BCL group, the NIF-BUT and Schemer I test scores were significantly improved at 1 month after operation compared with the control group (*P* = 0.012 and *P* < 0.001, respectively). Levels of IL-6, IL-8 and ICAM-1 were significantly increased in the control group at 1 month after the operation (*P* = 0.005, *P* = 0.038 and *P* = 0.022, respectively), while there was no difference in the BCL group. The increase in the IL-6 level in the control group was significantly higher compared with that in the BCL group (*P* = 0.047). In DED patients, there were significant correlations between ocular surface parameters and inflammatory molecules.

**Conclusions:**

Cataract surgery could lead to the development or worsening of DED. The application of BCLs after cataract surgery could stabilize the ocular surface and tear film, improve the corneal healing and reduce the inflammation. Collectively, our findings suggested that proper use of BCLs after cataract surgery played an effective role in the management of DED.

**Trial registration:**

ClinicalTrials, NCT04100031. Registered 18 September 2019—retrospectively registered

## Background

Cataract surgery remains one of the most effective interventions to improve vision and quality of life. However, many patients are still dissatisfied with the postoperative dry eye symptoms, which disturb their daily life. Various studies have demonstrated that cataract surgery may lead to the development and progression of dry eye disease (DED) [1–3]. DED is regarded as a multifactorial disorder, which can cause several ocular symptoms of discomfort, visual disturbance and tear film instability with potential damage to the ocular surface [4]. The possible mechanisms of postoperative DED are various, such as a reduction in tear production and corneal sensitivity, alteration in tear dynamics and stability, increased tear osmolarity, worsening of meibomian gland function, squamous metaplasia of conjunctiva or goblet cell loss [5–8]. The levels of inflammatory molecules, which play an important role in DED [9, 10], can also alter after cataract surgery, resulting in exacerbation of dry eye-related symptoms.

Since the nineteenth century, bandage contact lenses (BCLs) have been applied as a treatment for various anterior conditions that affect the ocular surface, including corneal erosions, corneal surface irregularity, corneal ulcers, dry eye and keratitis sicca [11]. They are also widely used after surgeries for their ability to relieve pain and promote corneal epithelial recovery [12, 13]. However, few studies have investigated the efficacy of BCLs after cataract surgery, especially in the management of dry eye-related symptoms.

In the present study, we assessed the pre- and postoperative dry eye-related symptoms, signs and inflammatory molecule levels of patients who underwent cataract surgeries, and also investigated the efficacy of BCLs in DED after the surgery. Our findings suggested that BCLs could be used as a therapeutic regimen to alleviate DED after cataract surgery.

## Materials and methods

This prospective study was approved by the Institutional Review Board of the Second Affiliated Hospital, Medical College of Zhejiang University, Hangzhou, China, and adhered to the tenets of the Declaration of Helsinki. Informed consent was obtained from all participants after a detailed explanation of the study. The clinical trial registration number of this study is NCT04100031.

### Study population and procedures

Patients who met the inclusion criteria were recruited in this study. The inclusion criteria were patients aged 60 years or older with age-related cataract and DED. Diagnosis criteria of DED [14] included a history of dry eye-related symptoms for at least 6 months and at least one of the following results: NIF-BUT ≤ 10 s, Schirmer I test score < 10 mm/5 min or meniscus height ≤ 0.35 mm. Exclusion criteria included a history of surgery and other ocular diseases, contact lens use or any ocular therapy, such as 0.05% cyclosporine A or steroids, in the last 3 months. Patients who had any systemic diseases were also excluded.

Patients were randomly divided into two groups before cataract surgery. Perioperative anti-inflammation and corticosteroids drops were prescribed routinely. Besides, the subjects in the BCL group were asked to wear BCLs (Bausch and Lomb PureVision2, balafilcon A, New York, USA) for 1 week from the first day after cataract surgery. Patients in the BCL group were excluded from the study if they lost the BCLs during this week.

### Patient evaluation

Eligible patients were evaluated for dry eye-related parameters at 3 days before cataract surgery (baseline), and at 1 week and 1 month after surgery. At the 1-week follow-up, for patients in the BCL group, the bandage contact lenses were removed at least 2 h before the exams. Clinical evaluations were performed in the following order: slit-lamp biomicroscopic examination, Ocular Surface Disease Index (OSDI), keratograph analysis and Schirmer I test. Physicians were uninformed to the group assignments.

The OSDI questionnaire was used to evaluate the dry eye-related symptoms of patients, which were graded on a scale of 0 to 4, and divided into three categories: (1) ocular symptom subscore (items 1 to 6); (2) vision-related function subscore (items 7 to 9); and (3) environmental-triggered subscore (items 10 to 12). Questions 4 and 5, which assessed the presence of blurred vision, were not administered to the participants as it was difficult to distinguish the symptom changes caused by cataract surgery alone or a combination of visual symptoms caused by cataract surgery-induced dry eye. The total OSDI score was calculated as follows: OSDI = [(sum of scores for all questions answered) × 100]/[(total number of questions answered) × 4] [15].

The keratography analysis was performed using a keratograph (Oculus, Wetzlar, Germany), which is useful in determining noninvasive keratograph tear meniscus height (NIKTMH), noninvasive first breakup time (NIF-BUT), noninvasive average breakup time (NIAvg-BUT), bulbar redness and meibography according to previously described methods [16, 17]. Redness was assessed and automatically scored by keratography based on the area percentage ratio of blood vessels in the bulbar conjunctiva. The score ranged from 0.0 to 4.0 (accurate to 0.1 unit). Meibography scores were obtained using the following grades for each eyelid based on the obstruction of the meibomian glands: 0 (no loss of meibomian glands); 1 (area loss less than one-third of the total meibomian gland area); 2 (area loss between one-third and two-thirds of the total meibomian gland area); and 3 (area loss more than two-thirds of the total meibomian gland area). The total meibography score was the sum of the scores of the upper and lower eyelids and recorded as 0 to 6.

The Schirmer I test was performed without anesthesia by placing one sterile strip (Schirmer Tear Test Strips, 5 × 35 mm; Liaoning Meizilin Pharmaceutical Co., Ltd., Liaoning, China) in the mid-lateral one-third of the inferior lid margin. Patients were asked to keep their eyes closed, and after 5 min, the length of wetting was determined in millimeters.

### Cataract surgery

After all examinations, patients were prescribed diclofenac sodium (Shenyang Xingqi Pharmaceutical Co., Ltd., Shenyang, China) eye drops four times per day until the surgery. Cataract surgeries were carried out by the same surgeon, who was uninformed to the group assignments until the surgery was completed. All surgical procedures were performed using standard equipment and identical methods for standard micro-incision phacoemulsification. Under topical anesthesia, all patients underwent a 2-mm clear corneal incision, capsulorhexis, phacoemulsification and intraocular lens implantation in the capsular bag. Subsequently, the corneal incision was sealed with stromal hydration. After the surgery, tobradex eye drops (Novartis, Basel, Switzerland) were administered to patients in both groups. There were no intraoperative complications in any case. Postoperatively, all patients were prescribed prednisolone (Allergan, Irvine, CA, USA) and levofloxacin (Shenyang Xingqi Pharmaceutical Co., Ltd., Shenyang, China) eye drops, four times per day for 1 week, and diclofenac sodium (Shenyang Xingqi Pharmaceutical Co., Ltd., Shenyang, China) eye drops four times per day for 1 month.

### Tear collection and multiplexed immunobead analysis

Tear samples were collected before other examinations. The collection was performed by the same technician to limit collection bias. To collect tear samples, 200 µL of normal saline was instilled into the inferior fornix. More than 100 µL of tear fluid and the buffer was collected with a micropipette at the lower lid near the lateral canthus. Topical anesthetics were not used during the tear collection. The tear samples were collected as soon as possible to minimize irritation to the ocular surface. The collected fluid was transferred to a 200-µL Eppendorf tube. The sample tube was placed on dry ice during the examination and subsequently stored at −80 °C until further analysis, and repeated freeze–thaw cycles were avoided. Inflammatory molecules were analyzed using Luminex commercial assays on a Bio-Plex 200™ System (Bio-Rad, Hercules, California, USA), including a multi-plex assay and a single-plex assay (Magnetic Luminex assay, R&D Systems, Minnesota, USA). The inflammatory molecules, including IL-1*β*, IL-6, chemokine (C-X-C motif) ligand 8 (CXCL8)/IL-8, IL-10, IL-12p70, TNF-*α* and ICAM-1, were analyzed. A total of 50 µL tear sample was used for each assay following the manufacturer’s protocols. Data were acquired and analyzed using the Bio-Plex Data Pro (Bio-Rad, Hercules, California, USA) that calculates molecular concentrations of tear inflammatory molecules using standard curves and a 5-parameter logistic curve-fitting model.

## Statistical analysis

To compare the sex and laterality between the BCL group and the control group, a Chi-square test was used. The Student’s t-test was used to compare mean age between the two groups. A linear mixed model with Bonferroni post hoc analysis was used to evaluate preoperative and postoperative clinical signs and symptoms. The Wilcoxon signed-rank test was used to compare inflammatory molecule levels between baseline and 1-month post-operation. The Mann–Whitney *U* test was used to compare inflammatory molecule levels between the two groups. Correlations between tear inflammatory molecules and clinical symptoms were analyzed using Spearman correlation. Data were analyzed using the SPSS for Mac 25.0 (SPSS Inc., Chicago, IL, USA). A P value less than 0.05 was considered statistically significant.

## Results

A total of 120 patients (140 eyes) were enrolled in this study. The BCL group included 70 eyes from 59 patients (30 females, 29 males, mean age ± standard deviation [SD], 75.07 ± 7.82 years; range, 60–91 years), and the control group included 70 eyes from 62 patients (32 females, 30 males, mean age ± SD, 75.67 ± 7.50 years; range, 61–94 years). Among the participants in the BCL group, there were no subjective reports of discomfort, irritation, blurred vision or elevated intraocular pressure during the use of BCLs. In addition, no adverse events (such as infection or inflammatory keratitis, conjunctivitis, redness, corneal abrasions, neovascularization or any other events) that could impede treatment were observed. Five patients were excluded because of lens loss in the first week after cataract surgery. Table 1 shows the patient characteristics. There were no significant differences in any parameters between the two groups before the surgery.

**Table 1:** Demographics and clinical features in both groups

Parameter	Control group	BCL group	*P* Value
Number of eyes	70	70	
OD (%)	37 (52.86%)	33 (47.14%)	0.306
Female (%)	32 (52.5%)	30 (50.8%)	0.502
Age (range)	75.67 ± 7.50 (61–94)	75.07 ± 7.82 (60–91)	0.674
NIKTMH (mm)	0.18 ± 0.08	0.18 ± 0.08	0.511
NIF-BUT (s)	4.37 ± 2.54	4.18 ± 2.33	0.654
NIAvg-BUT (s)	5.35 ± 3.04	5.07 ± 2.73	0.571
Redness score (0–4)	2.07 ± 0.46	2.03 ± 0.48	0.591
Meibography score (0–6)	3.59 ± 1.42	3.67 ± 1.43	0.729
Schirmer I test (mm)	7.11 ± 2.95	8.05 ± 4.67	0.175
OSDI (0–100)	20.35 ± 19.31	22.78 ± 23.04	0.511

*NIKTMH* noninvasive keratograph tear meniscus height; *NIF-BUT* noninvasive first breakup time; *NIAvg-BUT* noninvasive average breakup time; *OSDI* Ocular Surface Disease Index. Data are presented as means ± standard deviation (SD)

**Fig. 1:**
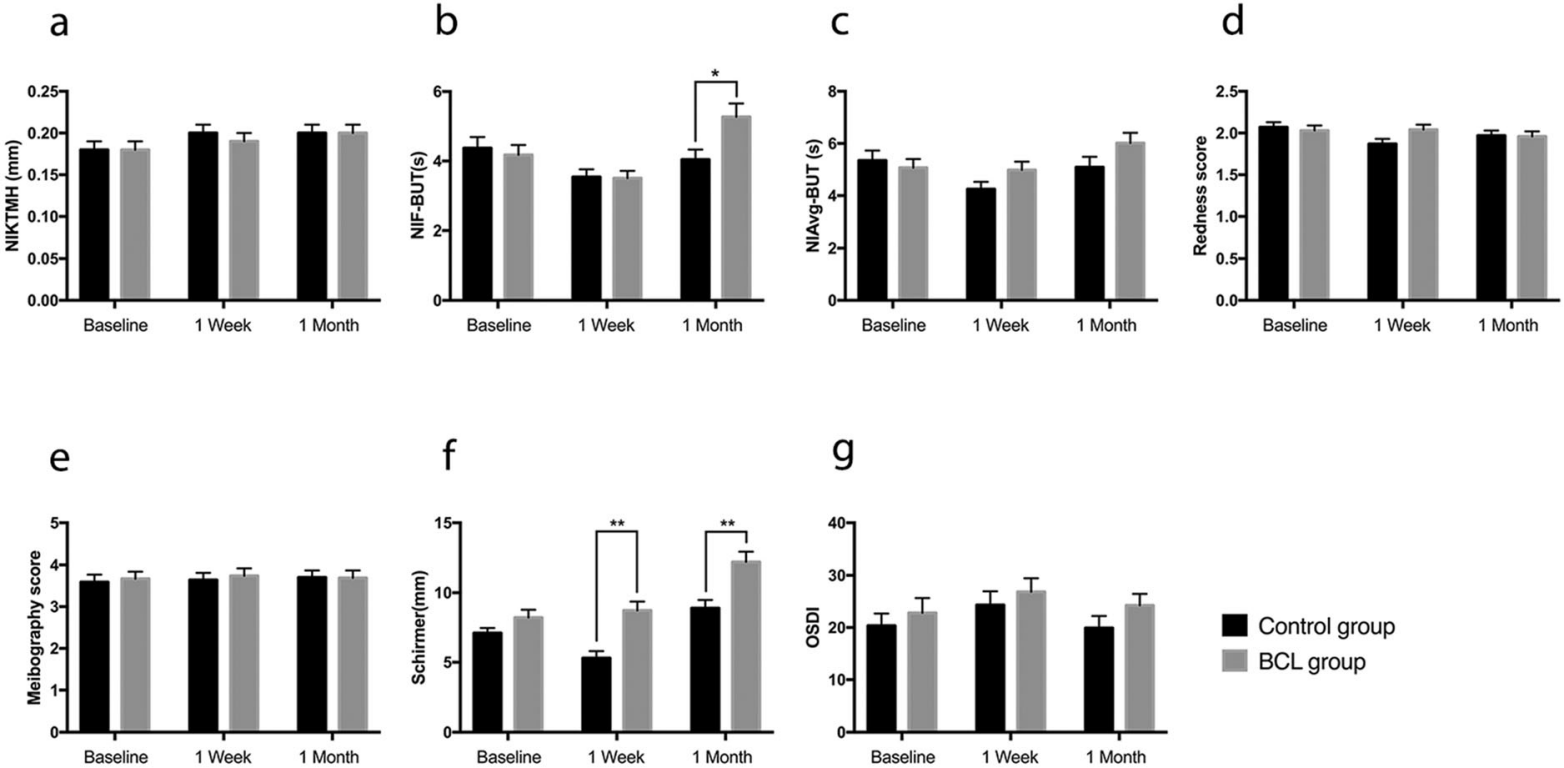
Changes in clinical signs and symptoms in the BCL and control groups after phacoemulsification. The clinical signs and symptoms were compared between the two groups at each time point. **a** Noninvasive keratography tear meniscus height (NIKTMH); **b** noninvasive first breakup time (NIF-BUT); **c** noninvasive average breakup time (NIAvg-BUT); **d** Bulbar redness; **e** Meibography score; **f** Schirmer I test; **g** Ocular Surface Disease Index (OSDI). Each value represents the mean ± standard error (SE). *P < 0.05, **P < 0 .01, using a linear mixed model with Bonferroni post hoc analysis

Table 2 details the results of preoperative and postoperative clinical signs and symptoms of both groups. In the linear mixed analysis, considering the interaction effect between the two groups and the three assessment time points, there were statistically significant differences in the measurement of NIf-BUT, bulbar redness and Schirmer I test score (*P* = 0.008, *P* = 0.047 and *P* = 0.013, respectively). In both the BCL and control groups, the mean NIF-BUT and NIAvg-BUT were decreased at 1 week after surgery and then increased again at 1 month after the surgery (Table 2). Compared with the baseline level, the mean value of NIAvg-BUT in the control group was significantly lower at 1 week after the surgery (*P* = 0.035), while there was no difference in the BCL group. Both the mean values of NIF-BUT and NIAvg-BUT in the BCL group at 1 month post-operation were significantly increased compared with those at 1 week post-operation and baseline (*P* < 0.001, *P* = 0.034, *P* = 0.006 and *P* = 0.024, respectively). However, in the control group, these parameters were not significantly different (Table 3). At 1 month post-operation, the mean NIF-BUT was significantly higher in the BCL group compared with the control group (*P* = 0.012) (Fig. 1b).

**Table 2:** Clinical signs and symptoms before and after cataract surgery in both groups

	Control group (70)			BCL group (70)			
Parameters	Baseline	1 Week	1 Month	Baseline	1 Week	1 Month	P Value^a^
NIKTMH	0.18 ± 0.01	0.20 ± 0.01	0.20 ± 0.01	0.18 ± 0.01	0.19 ± 0.01	0.20 ± 0.01	0.526
NIF-BUT	4.37 ± 0.32	3.54 ± 0.22	4.04 ± 0.29	4.18 ± 0.28	3.51 ± 0.21	5.27 ± 0.39	**0.008**
NIAvg-BUT	5.35 ± 0.38	4.25 ± 0.28	5.09 ± 0.4	5.07 ± 0.33	4.98 ± 0.32	6.02 ± 0.39	0.065
Redness score	2.07 ± 0.06	1.87 ± 0.06	1.97 ± 0.06	2.03 ± 0.06	2.04 ± 0.06	1.96 ± 0.06	**0.047**
Meibography score	3.59 ± 0.18	3.64 ± 0.17	3.70 ± 0.17	3.67 ± 0.17	3.74 ± 0.18	3.69 ± 0.18	0.805
Schirmer I test	7.11 ± 0.36	5.31 ± 0.50	8.89 ± 0.59	8.05 ± 0.57	8.73 ± 0.64	12.22 ± 0.74	**0.013**
OSDI	20.35 ± 2.34	24.32 ± 2.64	19.91 ± 2.29	22.78 ± 2.82	26.80 ± 2.6	24.20 ± 2.23	0.895

*NIKTMH* noninvasive keratography tear meniscus height; *NIF-BUT* noninvasive first breakup time; *NIAvg-BUT* noninvasive average breakup time; *OSDI* Ocular Surface Disease Index

Results are presented as means ± standard error (SE). ^a^*P* values are from linear mixed model with Bonferroni post hoc analysis considering the interaction effect between the 2 groups and the 3 time points. Significant differences are in bold

**Table 3:** Statistical analysis results of clinical signs and symptoms before and after cataract surgery in both groups

	*P* value^a^					
	Control group (70)			BCL group (70)		
	Baseline vs 1 W	Baseline vs 1 M	1 W vs 1 M	Baseline vs 1 W	Baseline vs 1 M	1 W vs 1 M
NIKTMH	0.064	0.079	> 0.99	> 0.99	0.431	> 0.99
NIF-BUT	0.053	> 0.99	0.525	0.066	**0.006**	** < 0.001**
NIAvg-BUT	**0.035**	> 0.99	0.149	> 0.99	**0.024**	**0.034**
Redness score	**0.016**	0.285	0.181	> 0.99	0.853	0.694
Meibography score	> 0.99	0.759	> 0.99	> 0.99	> 0.99	> 0.99
Schirmer I test	**0.009**	**0.032**	**< 0.001**	> 0.99	**< 0.001**	**< 0.001**
OSDI	0.697	> 0.99	0.616	0.508	> 0.99	> 0.99

*NIKTMH* noninvasive keratography tear meniscus height; *NIF-BUT* noninvasive first breakup time; *NIAvg-BUT* noninvasive average breakup time; *OSDI* Ocular Surface Disease Index. ^a^*P* values from linear mixed model with Bonferroni post hoc analysis. Significant differences are in bold

In the control group, the mean redness score was significantly decreased at 1 week after the surgery (*P* = 0.016). No significant difference was observed over time in the BCL group (Table 3). Although the mean redness score was higher in the BCL group compared with the control group at 1 week post-operation, such difference was not statistically significant (Fig. 1d).

In both study groups, tear secretion was significantly improved at 1 month after the surgery. In the control group, the mean Schirmer I test score was significantly lower at 1 week post-operation compared with that at baseline (P = 0.009). In contrast, no significant decrease in Schirmer I test score was noted in the BCL group (Table 3). Further, at both 1 week and 1 month after the surgery, the Schirmer I test score was significantly higher in the BCL group compared with the control group (*P* < 0.001 and *P* = 0.001, respectively; Fig. 1f). The NIKTMH, meibography score and OSDI did not alter significantly over time (Table 3), and no significant differences were observed between the two groups at either postoperative time point (Fig. 1a, e, g).

We randomly selected 50 eyes from each group to evaluate the concentrations of several inflammatory molecules in tears. The BCL group consisted of 50 eyes from 42 patients (21 females, 21 males, mean age ± SD, 74.39 ± 7.87 years; range, 60–91 years), and the control group consisted of 50 eyes from 45 patients (21 females, 24 males, mean age ± SD, 75.40 ± 7.03 years; range, 61–91 years). Table 4 lists the demographics and baseline inflammatory molecule levels of these patients. There were no significant differences between the two groups before cataract surgery.

**Table 4:** Patient demographics and baseline inflammatory molecule levels

	Control group	BCL group	*P* value
Number of eyes	50	50	
OD (%)	25 (50%)	27 (54%)	0.530
Age (range)	75.40 ± 7.03 (61–91)	74.39 ± 7.87 (60–91)	0.453
Female (%)	21 (46.67%)	21 (50%)	0.462
IL-1β	0.51 ± 0.66	0.53 ± 1.28	0.946
IL-6	0.92 ± 1.69	1.09 ± 1.1	0.128
Il-8	25.01 ± 34.03	26.6 ± 28.24	0.586
IL-10	0.3 ± 0.15	0.24 ± 0.11	0.065
IL-12	5.38 ± 1.37	5 ± 1.08	0.140
TNF-α	0.47 ± 0.48	0.52 ± 0.48	0.354
ICAM-1	1268.06 ± 748.21	1397.57 ± 696.74	0.332

*TNF* tumor necrosis factor; *IL* interleukin; *ICAM* intercellular cell adhesion molecule. Data are presented as means ± standard deviation (SD)

In the control group, the levels of IL-6, IL-8 and ICAM-1 were significantly increased at 1 month after the surgery compared with their baseline levels (*P* = 0.005, *P* = 0.038 and *P* = 0.022, respectively). However, in the BCL group, the levels of these inflammatory molecules did not significantly change from baseline to 1 month post-operation (Table 5). Moreover, the change in IL-6 level between baseline and 1 month post-operation in the BCL group was significantly smaller compared with that in the control group (*P* = 0.047) (Table 5). We also performed correlation analyses between the inflammatory molecules and clinical symptoms in all participants before cataract surgery (Table 6). The results showed that IL-6 and IL-8 were negatively correlated with NIF-BUT (*P* = 0.012 and *P* = 0.043, respectively), and IL-8 and IL-1*β* were negatively correlated with Schirmer I test score (*P* = 0.046 and *P* = 0.023, respectively).

**Table 5:** Difference in the inflammatory molecule levels before and at 1 month after cataract surgery in both groups

	Control group (*n* = 50)			BCL group (*n* = 50)			
	Baseline	1 month	*P* value^a^	Baseline	1 month	*P* value^a^	P value^b^
IL-1*β*	0.51 ± 0.66	0.85 ± 2.8	0.987	0.53 ± 1.28	0.77 ± 1.28	0.096	0.334
IL-6	0.92 ± 1.69	1.49 ± 1.64	**0.005**	1.09 ± 1.1	1.17 ± 1.32	0.823	**0.047**
IL-8	25.01 ± 34.03	40.3 ± 46.87	**0.038**	26.681.32	34.12 ± 41.99	0.451	0.370
IL-10	0.3 ± 0.15	0.31 ± 0.16	0.767	0.24 ± 0.11	0.29 ± 0.23	0.424	0.803
IL-12	5.38 ± 1.37	5.12 ± 2.27	0.245	5 ± 1.08	5.14 ± 1.66	0.648	0.214
TNF-*α*	0.47 ± 0.48	0.55 ± 0.45	0.128	0.52 ± 0.48	0.67 ± 0.75	0.457	0.607
ICAM-1	1268.06 ± 748.21	1584.54 ± 820.35	**0.022**	1397.57 ± 696.74	1624.06 ± 954.6	0.122	0.760

*TNF* tumor necrosis factor; *IL* interleukin; *ICAM* intercellular cell adhesion molecule. Data are presented as means ± standard deviation (SD). ^a^Wilcoxon signed-rank test between baseline and 1 month postoperatively in each group. ^b^Mann–Whitney *U* test between two groups in the change of inflammatory molecule levels from baseline to 1 month post-operation. Significant differences are in bold

**Table 6:** Correlation between tear inflammatory molecules and clinical symptoms in both groups before cataract surgery

		NIKTMH	NIF-BUT	NIAvg-BUT	Redness score	Meibography score	Schirmer I test
IL-1*β*	*r*	0.097	−0.175	−0.048	0.146	−0.085	−**0.276**
	*P* value	0.433	0.154	0.698	0.239	0.493	**0.023**
IL-6	*r*	0.03	**−0.257**	−0.169	0.079	0.151	−0.096
	*P* value	0.777	**0.012**	0.104	0.456	0.143	0.354
IL-8	*r*	−0.035	**−0.204**	−0.137	0.153	0.076	−**0.200**
	*P* value	0.731	**0.043**	0.176	0.136	0.453	**0.046**
IL-10	*r*	−0.037	0.096	0.123	−0.012	−0.003	−0.158
	*P* value	0.717	0.346	0.227	0.905	0.973	0.119
IL-12	*r*	−0.097	−0.079	−0.044	0.05	−0.064	−0.022
	*P* value	0.343	0.441	0.667	0.629	0.533	0.827
TNF-α	*r*	0.228	−0.105	−0.018	−0.048	−0.221	−0.153
	*P* value	0.056	0.382	0.88	0.696	0.062	0.197
ICAM-1	*r*	−0.094	−0.027	−0.104	0.003	0.108	0.152
	*P* value	0.375	0.795	0.326	0.98	0.303	0.146

*IL* interleukin; *TNF* tumor necrosis factor; *ICAM* intercellular cell adhesion molecule; *NIKTMH* noninvasive keratography tear meniscus height; *NIF-BUT* noninvasive first breakup time; *NIAvg-BUT* noninvasive average breakup time; *OSDI* Ocular Surface Disease Index. Spearman correlation coefficient (r) was used to analyze the associations between variables. Significant correlations are in bold

## Discussion

In the recent decade, silicone hydrogel has become one of the most popular BCL materials due to its high oxygen permeability, low water content, improved lens stability and centration, which facilitate corneal wound healing and enable extended wearing [18, 19]. Additionally, silicone hydrogel BCLs can also help to stabilize the tear film, promote the corneal healing and restore the normal cell turnover [20]. All of these characteristics provide a new therapeutic adjunct in the management of dry eye after cataract surgery. In this study, we investigated the safety and efficacy of BCLs for the management of DED after cataract surgery.

In this study, we observed a worsening of dry eye symptoms at 1 week and 1 month after cataract surgery in the control group. By using BCLs after the operation, we found an improvement in dry eye-related parameters at both these time points compared with the control group. Moreover, compared with the baseline, the dry eye-related symptoms were significantly improved at 1 month after the surgery. In the control group, NIF-BUT and NIAvg-BUT were decreased at 1 week post-operation and did not return to the baseline levels at 1 month post-operation. In contrast, the meibography score was not significantly changed over time in both groups. These results were consistent with the previous studies that the meibomian gland function can be altered after cataract surgery without obvious morphological changes [1]. In the BCL group, the NIF-BUT and NIAvg-BUT were significantly increased at 1 month post-operation. The use of BCLs aims to maximize the lubrication and tear function of the eye while maintaining the integrity of the ocular surface. It can also help lock water and prevent the tear film from evaporating. However, we found that NIF-BUT might not have clinical significance. Nevertheless, the results could still provide some meaningful recommendations for the use BCL in the management of dry eye disease after cataract surgery.

According to our results, wearing BCLs for an extended period did not induce tear deficiency. The Schirmer test score has been reported to either decrease [5] or remain unaltered after cataract surgery [1]. The Schirmer I test score at 1 week and 1 month post-operation in the BCL group was significantly higher compared with the control group. One possible explanation for this observation might be the recovery of corneal sensation. Cataract surgery procedures can disrupt the normal organization of corneal innervation, which results in impaired epithelial wound healing, increased epithelial permeability, decreased epithelial metabolic activity and loss of cytoskeletal structures [21]. The recovery time appears to be related to the size of the corneal wound, and the decrease in corneal sensitivity with the subsequent reduction in tear production may bring about aqueous tear deficient dry eye [22, 23]. It is widely accepted that BCLs are commonly used to reduce the healing time of the corneal epithelium [13]. In addition, several studies have shown that wearing silicone hydrogel lenses can reduce dryness and discomfort [24, 25]. Our results indicated that early application of BCL after cataract surgery could improve dry eye symptoms, and this improvement might be secondary to better corneal wound healing and increased tear film stability.

Previous studies have shown that the bulbar redness is a reaction of the blood vessels to the contact lens [26, 27]. Both surgery and DED can induce bulbar redness. The difference in redness score was not statistically different between the two groups. These results indicated that there were no significant signs or symptoms of conjunctival congestion after extended wearing of BCLs. OSDI is a questionnaire to subjectively assess dry eye-related symptoms. Our results indicated that there was a lack of association between signs and symptoms in DED patients.

We observed that the level of IL-8, IL-6 and ICAM-1 was significantly increased after cataract surgery in the control group, while no significant changes in the BCL group were observed. Moreover, ocular surface parameters were significantly correlated with inflammatory molecules. It has been reported that the level of IL-6, IL-8 and ICAM-1 is increased in tears of DED patients [28–30]. These results suggest that inflammation plays a key role in the pathogenesis of DED. It is well known that the stimulation of the ocular surface during cataract surgery can cause an inflammatory response that in turn stimulates and destroys the ocular surface structure, leading to excessive evaporation of the tear film, and this becomes a vicious circle [31].

The proinflammatory cytokines, such as IL-1*β*, IL-6 and TNF-*α*, are increased due to inflammatory and mechanical damages. IL-6 has been recognized as one of the most important molecules in DED. Previous studies have shown that the IL-6 level is significantly increased in tear samples of DED patients, and it has a significant correlation with various ocular parameters [32, 33]. Our results were similar to these studies. The significant increase in the levels of tear inflammatory molecules in the control group suggested that the induction of ocular surface inflammation resulted in a deterioration in postoperative DED. The application of BCLs reduces the stimulation of the cornea by the eyelids, and the vulnerable or healing epithelium is protected, which helps the corneal epithelium to maintain its stability. At the same time, it can also mechanically prevent microorganisms from entering the corneal stroma, thus blocking microbial invasion. In addition, the BCL has good penetrating properties and consistent drug delivery [34] for eye drops, such as antibiotics and steroids, promoting the healing of the epithelium. Altogether, these factors reduce the inflammation of the ocular surface and alleviate the DED. Early use of BCL requires strict aseptic operation, good compliance and close follow-up attention. Otherwise, it may increase the risk of postoperative infection.[35]

The main limitation of this study is the short follow-up time. Moreover, only eight inflammatory molecules were analyzed in this study, and some important molecules might have been neglected. Therefore, more inflammatory molecules and more clinical parameters need to be studied in the future.

## Conclusions

Our study revealed that routine cataract surgery led to the development or worsening of DED. The application of BCLs after cataract surgery could notably stabilize the ocular surface and tear film, improve the corneal healing and reduce the inflammation. Besides, BCLs were easy to fit in and comfortable for long-term wear. However, early use of BCL after cataract surgery requires more strict aseptic operation by physicians and better patient compliance. Collectively, our findings suggested that proper use of BCL after cataract surgery played an effective role in the management of DED.

## Data Availability

All data generated and analyzed during this study are included in this article.
